# Enamel Remineralization Efficacy of Coconut Milk and Lyophilized Coconut Extract in Different Concentrations on Demineralized Enamel Surfaces: An In-Vitro Study

**DOI:** 10.7759/cureus.44712

**Published:** 2023-09-05

**Authors:** Nivethigaa Balakrishnan, Aravind Kumar Subramanian, Rajalakshmanan Eeswaramoorthy, Dhireka Kandasamy

**Affiliations:** 1 Orthodontics and Dentofacial Orthopedics, Saveetha Dental College and Hospitals, Saveetha Institute of Medical and Technical Sciences, Saveetha University, Chennai, IND; 2 Biomaterials, Saveetha Dental College and Hospitals, Saveetha Institute of Medical and Technical Sciences, Saveetha University, Chennai, IND

**Keywords:** lyophilized coconut extract, coconut milk, ph cycling, artificial carious lesions, white spot lesions, enamel remineralization

## Abstract

Aim

The current study's objective is to determine the remineralizing efficacy of freeze-dried lyophilized coconut extract and coconut milk made from freshly grated coconut on artificial carious lesions produced by pH cycling.

Materials and methods

Freshly extracted coconut pulp was split into two parts. The first half was blended to obtain coconut milk, and the second part was freeze-dried and lyophilized. Tooth slabs were prepared from extracted third molar teeth. After being demineralized for 72 hours, the tooth samples were remineralized by submerging them in the appropriate remineralizing solution, which is as follows: Group 1 received 25 mL of the Remineralization solution (the control); Group 2 received 2.5 g of coconut milk and 25 mL of the solution (1:1); and Group 3 received 5 g of coconut milk and 25 mL of the solution (2:1). 2.5 g of freeze-dried, lyophilized coconut extract was given to Group 4 along with 25 mL of remineralization solution (1:1), and 5 g of freeze-dried, lyophilized coconut extract was given to Group 5 along with 25 mL of remineralization solution (2:1). Microhardness and contact angle measurements were made. An Excel spreadsheet was filled up with values from after demineralization, and after remineralization. A statistical analysis was performed using SPSS 26.0 (IBM Corp., Armonk, NY). Using descriptive statistics, the pretreatment mean values for the microhardness and contact angle of the various groups were evaluated. Post-hoc Tukey tests were utilized to compare the analytic results of the various groups.

Results

Among the various concentrations of freshly extracted coconut milk, the contact angle in concentrations of 1:1 was 81.22 ± 1.62 deg, and that in concentrations of 2:1 was 88.01 ± 1.85 deg. Between the two concentrations of the lyophilized coconut extract group, the contact angle in 1:1 was 75.05 ± 2.29 deg, and in 2:1 was 71.37 ± 0.85 deg. In the coconut milk group, the value of microhardness was 261 ± 6.4 kg/cm^2^ at a lower concentration and 322 ± 3.9 kg/cm^2^ at a higher concentration. In the lyophilized coconut group, the lower concentration exhibited a microhardness of 211 ± 7.2 kg/cm^2^, whereas in the higher concentration, it was 324 ± 4.04 kg/cm^2^.

Conclusion

Of the various concentrations of coconut milk and lyophilized coconut used, coconut milk at a higher concentration exhibits the highest contact angle, and the latter at a higher concentration exhibits the lowest contact angle. In both groups, high concentrations of the material exhibited high microhardness values compared to lower concentrations of the same.

## Introduction

Enamel, the outer covering of teeth, must tolerate a variety of chemical and physical stresses. Compressive stresses, abrasion, attrition, and, most significantly, acidic challenges from plaque and diet are among them [1]. In an oral environment, plaque fluid and saliva are in close contact with the outermost part of dental enamel, and the surfaces of the enamel HAP crystals are in dynamic equilibrium with these nearby watery phases. The concentration of calcium and phosphate ions in the solution, as well as pH, affect the rate and amount of mineral dissolution in the enamel [2].

After orthodontic fixed appliances are placed in the oral cavity, there is a significant shift in the bacterial ecology, with larger concentrations of acidogenic bacteria like Streptococcus mutans and Lactobacillus. If these bacteria have access to enough fermentable carbohydrates, they will make acidic byproducts that will decrease the pH of the plaque. Carious decalcification happens when the pH falls below the remineralization threshold. A WSL represents the earliest clinical sign of this demineralization. Such lesions have been clinically produced in a period of four weeks, which corresponds to the interval between two orthodontic appointments [3,4].

The various available modalities for prevention include the use of fluoride-rich rinses or pastes, tooth creams, and rinses that increase the oral availability of Ca and P ions, which are essential for maintaining a balance in the dissolution and remineralization of dental enamel [5-8].

Coconut, or Cocos nucifera, is one of the fundamental raw ingredients in a typical home. In the food sector, coconut is used in a huge variety of goods. This has excellent nutritive value, with higher amounts of vitamins and minerals essential for the general health of the body. The major minerals found in raw coconut milk are Phosphorus, calcium, and Potassium. Also, the amount of calcium was the highest, which was estimated to be about 800 mg [9].

Previous studies on coconut have shown its excellent remineralizing effects [10]. Despite having many health benefits in various forms [11], milk made from freshly grated coconut pulp has been shown to have fewer drawbacks, such as a relatively short shelf life that makes it challenging to use for extended periods of time. In order to prevent deterioration, efforts have been undertaken to increase the quality of the newly manufactured coconut milk [12].

Though many products were tested previously, the occurrence of white spot lesions still remains. This study was done with an interest to identify the possible benefits of using extracts from coconut, prepared by two different methods and concentrations, on the management of white spot lesions. The current study's objective is to determine the remineralizing efficacy of freeze-dried lyophilized coconut extract and coconut milk made from freshly grated coconut on artificial carious lesions produced by pH cycling.

## Materials and methods

Sample preparation

This study was performed at a university setting in the research facility at Saveetha Dental College and Hospitals, India. Coconut obtained from a fresh source in the Tirupur district of Tamil Nadu, India, was used for the purpose of the study. Freshly extracted coconut pulp was grated, and the quantity was equally split into two equal parts (200 g each). One-half of the sample was used for the extraction of the milk using a blender and cheesecloth. Following the extraction of the coconut milk, the sample was stored at −20 °C to prevent any spoilage of the material. The other half of the sample was freeze-dried at −80 degrees, lyophilized for 48 hours, and then stored in a refrigerator. An artificial saliva solution was prepared in the laboratory [13-15].

Demineralization solution preparation

A demineralization solution was prepared by dissolving 323.4 g of calcium chloride (CaCl_2_), 400 mL of acetic acid (CH_3_COOH), and 300 mL of monobasic potassium phosphate (KH_2_PO_4_). The pH of the solution was adjusted from 4.4 to 4.7 using a 2N sodium hydroxide (NaOH) solution and a 1N hydrochloric acid (HCl) solution [13-15].

Remineralization solution preparation

A remineralization solution was prepared by dissolving 156 g of dipotassium hydrogen orthophosphate K_2_HPO_4_, 11.184 g of potassium chloride KCl, and 220.53 g of calcium chloride CaCl_2_. The pH of the solution was adjusted to 7 [13-15]. To identify any changes in remineralizing efficacy with changing concentration, 1:1 and 2:1 concentrations of both extracts were tested.

Following this, various solutions were prepared which are as follows: Group 1: 25 mL of remineralization solution (control), Group 2: 2.5 g of coconut milk with 25 mL of remineralization solution (1:1), Group 3: 5 g of coconut milk with 25 mL of remineralization solution (2:1), Group 4: 2.5 g of freeze-dried, lyophilized coconut extract with 25 mL of remineralization solution (1:1), Group 5: 5 g of freeze-dried, lyophilized coconut extract with 25 mL of remineralization solution (2:1). After preparation, all of these were kept at room temperature.

Tooth slab preparation 

**Figure 1:**
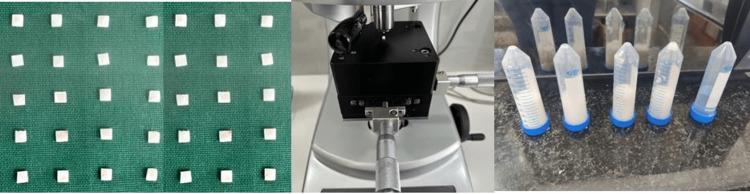
Tooth slab preparation measuring 3*3*1.5 (left), microhardness value assessed using a Vickers Microhardness tester (middle), and sample preparation for remineralization (right).

Extracted third molar teeth were obtained, and scaling was done to remove any debris on the tooth surface. From each of the tooth samples, square-shaped tooth slabs measuring 3*3*1.5 mm (3 mm length, 2 mm breadth, and 1.5 mm depth) were obtained (Figure 1). To ensure an equal sample size in all groups block randomization was employed. Hence seven samples were allocated to each of the five groups (one control and four treatment groups) [13-15]. 

Remineralization procedure

The samples (seven in each group) were immersed in the demineralization solution for 72 hours. After this, the tooth slabs were collected, washed using a deionized water solution, and immersed in the corresponding remineralization solution for 14 days (Figure 1). 

Microhardness

The Vickers micro-hardness tester (Shimadzu Model: HMV-G Microhardness Tester) was used to gauge each specimen's enamel's surface hardness. Vickers diamond indenters were used to make the indentations, which were made at a weight of HV 0.3 (2.942 N) with a dwell period of twenty seconds. A 40x magnification was used to photograph the indentation (Figure 1).

Contact angle

Using a Contact Angle Goniometer (Ossila), the contact angle of the prepared sealer materials was measured using the sessile drop method. By dropping 5 μL of water onto the dry sealer material samples at five separate locations, contact angle readings were taken. Readings of the average contact angle were reported.

Statistical analysis

Values from before treatment, after demineralization, and after remineralization was entered into an Excel spreadsheet. Statistical analysis was then carried out (using IBM SPSS Statistics for Windows, Version 26.0. Released 2019. IBM Corp., Armonk, NY). The mean values for the microhardness and contact angle among the various groups before pretreatment were examined using descriptive statistics. To compare the analytical outcomes of the various groups, post-hoc Tukey tests were used.

## Results

Contact angle

**Figure 2:**
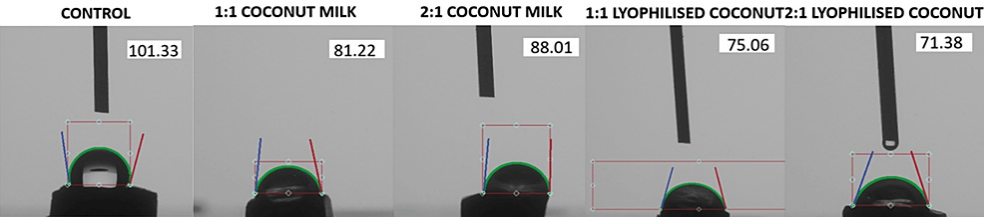
Contact angle measurements among the various groups made using a Goniometer (Oscillo).

The mean values of the contact angle among the various groups are mentioned in Table 1. The control group treated with the remineralization solution alone had a contact angle of 101.30 ± 1.51 deg. Among the various concentrations of freshly extracted coconut milk, the contact angle in concentrations of 1:1 was 81.22 ± 1.62 deg, and that in concentrations of 2:1 was 88.01 ± 1.85 deg. Between the two concentrations of the lyophilized coconut extract group, the contact angle in 1:1 was 75.05 ± 2.29 deg, and in 2:1 was 71.37 ± 0.85 deg. An ANOVA test was done to check the statistical significance of the results. The mean difference is significant at the 0.05 level. Table 2 exhibits a post-hoc Tukey analysis among the groups (Figure 2).

**Table 1:** Descriptive statistics for the measurement of contact angle among the various control and treatment groups. * P <0.05 Statistically significant.

	N	Minimum	Maximum	Mean (Degree)	Std. Deviation	Sig.
Demin	7	89.20	91.80	90.9429	0.98464	< .001*
Control	7	98.90	103.40	101.3000	1.51548	
1:1 C Milk	7	78.10	83.40	81.2214	1.62836	
1: 1 LYOPHILIZED COCONUT	7	70.20	77.30	75.0571	2.29118	
2:`1 C Milk	7	85.70	91.20	88.0143	1.85870	
2: 1 LYOPHILIZED COCONUT	7	70.30	72.90	71.3786	0.85238	

**Table 2:** Post-hoc Tukey test among the various control and treatment groups for the contact angle testing. * The mean difference is significant at the 0.05 level.

(I) Groups	(J) Groups	Mean Difference (I-J) (Degree)	Std. Error	Sig.	95% Confidence Interval	
					Lower Bound	Upper Bound
Demin	Control	-10.35714^*^	.85492	< 0.001*	-12.9292	-7.7851
	1:1 C Milk	9.72143^*^	.85492	<0.001*	7.1493	12.2935
	1:1 Lyophilized coconut	15.88571^*^	.85492	< 0.001*	13.3136	18.4578
	2:1 C Milk	2.92857^*^	.85492	0.018*	.3565	5.5007
	2:1 Lyophilized coconut	19.56429^*^	.85492	< 0.001*	16.9922	22.1364
Control	Demin	10.35714^*^	.85492	<0.001*	7.7851	12.9292
	1:1 C Milk	20.07857^*^	.85492	< 0.001*	17.5065	22.6507
	1:1 Lyophilized coconut	26.24286^*^	.85492	<0.001*	23.6708	28.8149
	2:1 C Milk	13.28571^*^	.85492	< 0.001*	10.7136	15.8578
	2:1 Lyophilized coconut	29.92143^*^	.85492	< 0.001*	27.3493	32.4935
1:1 C Milk	Demin	-9.72143^*^	.85492	< 0.001*	-12.2935	-7.1493
	Control	-20.07857^*^	.85492	< 0.001*	-22.6507	-17.5065
	1:1 Lyophilized coconut	6.16429^*^	.85492	<0.001*	3.5922	8.7364
	2:1 C Milk	-6.79286^*^	.85492	< 0.001*	-9.3649	-4.2208
	2:1 Lyophilized coconut	9.84286^*^	.85492	< 0.001*	7.2708	12.4149
1:1 Lyophilized coconut	Demin	-15.88571^*^	.85492	< 0.001*	-18.4578	-13.3136
	Control	-26.24286^*^	.85492	< 0.001*	-28.8149	-23.6708
	1:1 C Milk	-6.16429^*^	.85492	< 0.001*	-8.7364	-3.5922
	2:1 C Milk	-12.95714^*^	.85492	< 0.001*	-15.5292	-10.3851
	2:1 Lyophilized coconut	3.67857^*^	.85492	0.002*	1.1065	6.2507
2:1 C Milk	Demin	-2.92857^*^	.85492	0.018*	-5.5007	-.3565
	Control	-13.28571^*^	.85492	< 0.001*	-15.8578	-10.7136
	1:1 C Milk	6.79286^*^	.85492	< 0.001*	4.2208	9.3649
	1:1 Lyophilized coconut	12.95714^*^	.85492	< 0.001*	10.3851	15.5292
	2:1 Lyophilized coconut	16.63571^*^	.85492	< 0.001*	14.0636	19.2078
2:1 Lyophilized coconut	Demin	-19.56429^*^	.85492	< 0.001*	-22.1364	-16.9922
	Control	-29.92143^*^	.85492	< 0.001*	-32.4935	-27.3493
	1:1 C Milk	-9.84286^*^	.85492	< 0.001*	-12.4149	-7.2708
	1:1 Lyophilized coconut	-3.67857^*^	.85492	0.002*	-6.2507	-1.1065
	2:1 C Milk	-16.63571^*^	.85492	< 0.001*	-19.2078	-14.0636

Microhardness

Microhardness testing was performed using a Vickers microhardness tester. The mean values of the microhardness values on the enamel surfaces of the tooth slabs are mentioned in Table 3. In the control group, the value was 259 ± 1.91 kg/cm^2.^ In the coconut milk group, the value of microhardness was 261 ± 6.4 kg/cm^2^ at a lower concentration and 322 ± 3.9 kg/cm^2 ^at a higher concentration. In the lyophilized coconut group, the lower concentration exhibited a microhardness of 211 ± 7.2 kg/cm^2^, whereas in the higher concentration, it was 324 ± 4.04 kg/cm^2^. An ANOVA test was done to check the statistical significance of the results. The mean difference is significant at the 0.05 level. Table 4 exhibits a post-hoc Tukey analysis among the groups.

**Table 3:** Descriptive statistics for the measurement of microhardness among the various control and treatment groups. * P <0.05 Statistically significant.

	N	Minimum	Maximum	Mean (kg/cm^2^)	Std. Deviation	Sig.
Demin	7	297.00	303.00	300.0000	2.38048	<0.001*
Control	7	256.00	261.00	259.0000	1.91485	
1:1 Coconut milk	7	250.00	270.00	261.0000	6.48074	
1:1 Lyophilized coconut	7	201.00	219.00	211.0000	7.28011	
2:1 Coconut milk	7	316.00	329.00	322.0000	3.91578	
2:1 Lyophilized coconut	7	320.00	330.00	324.0000	4.04145	

**Table 4:** Post-hoc Tukey test among the various control and treatment groups for the microhardness testing. * The mean difference is significant at the 0.05 level.

(I) Groups	(J) Groups	Mean Difference (I-J) (kg/cm^2^)	Std. Error	Sig.	95% Confidence Interval	
					Lower Bound	Upper Bound
Demin	Control	41.00000^*^	2.54484	<0.001*	33.3437	48.6563
	1:1 Coconut milk	39.00000^*^	2.54484	<0.001*	31.3437	46.6563
	1:1 Lyophilized coconut	89.00000^*^	2.54484	<0.001*	81.3437	96.6563
	2:1 Coconut milk	-22.00000^*^	2.54484	<0.001*	-29.6563	-14.3437
	2:1 Lyophilized coconut	-24.00000^*^	2.54484	<0.001*	-31.6563	-16.3437
Control	Demin	-41.00000^*^	2.54484	<0.001*	-48.6563	-33.3437
	1:1 Coconut milk	-2.00000	2.54484	0.968	-9.6563	5.6563
	1:1 Lyophilized coconut	48.00000^*^	2.54484	<0.001*	40.3437	55.6563
	2:1 Coconut milk	-63.00000^*^	2.54484	<0.001*	-70.6563	-55.3437
	2:1 Lyophilized coconut	-65.00000^*^	2.54484	<0.001*	-72.6563	-57.3437
1:1 Coconut milk	Demin	-39.00000^*^	2.54484	<0.001*	-46.6563	-31.3437
	Control	2.00000	2.54484	0.968	-5.6563	9.6563
	1:1 Lyophilized coconut	50.00000^*^	2.54484	<0.001*	42.3437	57.6563
	2:1 Coconut milk	-61.00000^*^	2.54484	<0.001*	-68.6563	-53.3437
	2:1 Lyophilized coconut	-63.00000^*^	2.54484	<0.001*	-70.6563	-55.3437
1:1 Lyophilized coconut	Demin	-89.00000^*^	2.54484	<0.001*	-96.6563	-81.3437
	Control	-48.00000^*^	2.54484	<0.001*	-55.6563	-40.3437
	1:1 Coconut milk	-50.00000^*^	2.54484	<0.001*	-57.6563	-42.3437
	2:1 Coconut milk	-111.00000^*^	2.54484	<0.001*	-118.6563	-103.3437
	2:1 Lyophilized coconut	-113.00000^*^	2.54484	<0.001*	-120.6563	-105.3437
2:1 Coconut milk	Demin	22.00000^*^	2.54484	<0.001*	14.3437	29.6563
	Control	63.00000^*^	2.54484	<0.001*	55.3437	70.6563
	1:1 Coconut milk	61.00000^*^	2.54484	<0.001*	53.3437	68.6563
	1:1 Lyophilized coconut	111.00000^*^	2.54484	<0.001*	103.3437	118.6563
	2:1 Lyophilized coconut	-2.00000	2.54484	0.968	-9.6563	5.6563
2:1 Lyophilized coconut	Demin	24.00000^*^	2.54484	<0.001*	16.3437	31.6563
	Control	65.00000^*^	2.54484	<0.001*	57.3437	72.6563
	1:1 Coconut milk	63.00000^*^	2.54484	<0.001*	55.3437	70.6563
	1:1 Lyophilized coconut	113.00000^*^	2.54484	<0.001*	105.3437	120.6563
	2:1 Coconut milk	2.00000	2.54484	0.968	-5.6563	9.6563

## Discussion

Remineralization efficacy was assessed using contact angle, wettability, and microhardness, which are measures of the physical properties of a material. The contact angle determines how well a liquid can wet a solid's surface. The shape that a drop will take on a surface is determined by the surface tension of the fluid and the properties of the surface. If the liquid distributes evenly throughout the solid surface, a contact angle of 0° implies full wetting, whereas a range of 0° to 90° denotes a wettable hydrophilic surface. The substance becomes hydrophobic and repellent after 90 degrees.

In the current investigation, the wettability of the demineralized enamel surface shows that the material exhibits borderline hydrophobic behavior. In the current investigation, the control solution's mean contact angle value ranged from 98° to 101°, showing a less wettable nature. The treatment groups, in contrast, exhibited a less hydrophilic to almost hydrophobic nature. Comparing freshly extracted coconut milk and lyophilized coconut extract, a 2:1 concentration of the former exhibits a more hydrophobic nature. Also, studies have shown that materials that exhibit a hydrophobic nature are resistant to bacterial adhesion and acid attack. In our case, all the materials exhibit the least hydrophilicity, indicating that they are least vulnerable to bacterial adhesion and further demineralization [16].

Previous studies have shown that the mean contact angle of the topical fluoride APF gel on the anterior tooth surface is greater when compared to the posterior tooth surface. This signifies that greater wettability is seen on posterior tooth surfaces and the posterior tooth surfaces are prone to WSL [17]. In a study by Morales et al., an increase in the hydrophobic nature of the material was seen after treatment with various remineralization agents [18].

The microhardness values of the enamel surface signify the quality of the remineralization on the artificially created white spot lesions. Specific areas of the sample can be tested using this method. This is a load- and time-dependent property. Following microhardness testing with the Vickers Microhardness tester, it was determined that the enamel surface of the demineralized enamel exhibited a mean hardness value of 300 MPa. Comparing the varying concentrations of the material tested, the higher concentration of the material exhibited a higher hardness value. Between the two extraction methods used, Lyophilized coconut extract had an increase in the mean values of microhardness compared to coconut extract.

Previously, a comparison of the effects of several remineralizing toothpastes was studied, and it was shown that the hydroxyapatite-based toothpaste significantly increased the microhardness values [19]. In another study, it was shown that toothpaste made of CCP-ACP had a better improvement in mean hardness [20]. Also, Changes in the microhardness values were noted after bleaching treatment on the enamel surface. Of the various materials tested, sodium chlorite-containing bleaching agents were shown to have reduced the microhardness of the enamel surface [21-23]. Another study by Rafiei et al. showed the potential for re-hardening WSLs in deep layers of enamel by combining the application of a CO_2_ laser with remineralizing paste when the laser is irradiated before the paste [24].

Limitations of the study

This study was done under controlled conditions in a research laboratory. Hence confounding patient factors could not be considered. After preparation of oral rinses/tooth creme consisting of these extracts, the material will be subjected to further laboratory testing followed by evaluation of the in vivo clinical efficiency of the same.

## Conclusions

Of the various concentrations of coconut milk and lyophilized coconut used, coconut milk at a higher concentration, and lyophilized coconut at a lower concentration exhibit the highest contact angles, indicating a more acid-resistant behavior of these remineralized surfaces. Assessing the microhardness values of the remineralized enamel among both groups, high concentrations of the material exhibited high post-treatment microhardness values compared to lower concentrations of the same.
